# #AMRrounds: a systematic educational approach for navigating bench to bedside antimicrobial resistance

**DOI:** 10.1093/jacamr/dlad097

**Published:** 2023-08-14

**Authors:** Elaine Liu, Andrea M Prinzi, Jovan Borjan, Samuel L Aitken, Patricia A Bradford, William F Wright

**Affiliations:** Division of Pharmacy and Division of Infectious Diseases, The Johns Hopkins Bayview Medical Center, 5200 Eastern Avenue, Baltimore, MD, USA; US Medical Affairs, bioMérieux, Salt Lake City, UT 84104, USA; Division of Pharmacy, The University of Texas MD Anderson Cancer Center, Houston, TX, USA; Department of Pharmacy, Michigan Medicine, Ann Arbor, MI, USA; Antimicrobial Development Specialists LLC, Nyack, NY, USA; Division of Infectious Diseases, Department of Medicine, Johns Hopkins University School of Medicine, 733 North Broadway, Baltimore, MD, USA

## Abstract

Antimicrobial resistance (AMR) continues to serve as a major global health crisis. Clinicians practising in this modern era are faced with ongoing challenges in the therapeutic management of patients suffering from antimicrobial-resistant infections. A strong educational understanding and synergistic application of clinical microbiology, infectious disease and pharmacological concepts can assist the adventuring clinician in the navigation of such cases. Important items include mobilizing laboratory testing for pathogen identification and susceptibility data, harnessing an understanding of intrinsic pathogen resistance, acknowledging epidemiological resistance trends, recognizing acquired AMR mechanisms, and consolidating these considerations when constructing an ideal pharmacological plan. In this article, we outline a novel framework by which to systematically approach clinical AMR, encourage AMR-related education and optimize therapeutic decision-making in AMR-related illnesses.

## Introduction

One of the biggest concerns to public health in the twenty-first century is antimicrobial resistance (AMR), which continues to be a global crisis.^1–5^ A wide range of organizations and academics concur that AMR requires a comprehensive, coordinated action plan to combat.^2–5^ If the problem is not adequately addressed by 2050, it is predicted that AMR-related fatalities could affect up to 10 million people annually and cost the world economy up to $100 trillion dollars.^5^

Antimicrobial stewardship programmes (ASPs) have been implemented in various contexts to improve the use of antibiotics, postpone the emergence of resistance, and maintain patient safety while limiting additional healthcare costs.^5^ According to the most recent research, ASPs have been linked to a 10% drop in antibiotic prescriptions and a 28% drop in antibiotic consumption.^5^ A 28% decrease in antibiotic use among WHO ‘watch’ group antibiotics has also been connected to ASPs.^5^ Many recent studies have also demonstrated the effectiveness of the traditional physician–pharmacist and primarily pharmacist-driven ASPs on lowering antibiotic consumption rates, the occurrence of AMR-related illnesses, and healthcare expenditures.^5–10^ Ultimately, ASPs serve to improve antimicrobial use in a variety of settings. Importantly, leveraging knowledge of how to optimize antimicrobial use in an increasingly resistant world (both on an individual case scale as well as informing larger institutional education and practice) is critical in the successful accomplishment of this goal.

**Figure 1.**
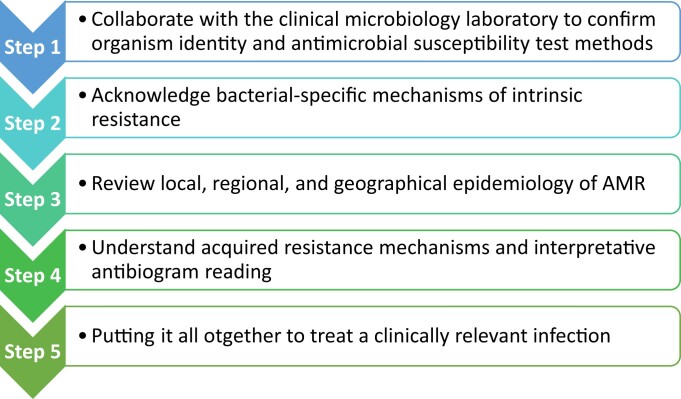
The five fundamental principles of AMRrounds.

Developing a clinically useful approach to addressing AMR is an integral component of ASPs globally.^11^ AMR rounds (AMRrounds) was a programme developed by one author (W.F.W.) as a standardized, systematic approach to addressing AMR within the context of a larger ASP program at Johns Hopkins. It was developed primarily as an educational tool for teaching core concepts of AMR to students, residents and fellows involved in infectious diseases management. While originally crafted with academic tenants in mind, the five main principles of the AMRrounds (Figure 1) approach integrate concepts from clinical infectious disease, clinical laboratory microbiology and clinical pharmacy practice to further provide a methodical perspective that clinicians can apply from start to finish in the care of patients with concern for AMR infections.

Recently, the AMRrounds approach was shared on social media (e.g. Twitter, #AMRrounds) and garnered attention after clinical microbiologists, PharmDs and infectious disease clinicians began to share susceptibility reports containing drug MICs and associated interpretations as either susceptible, intermediate or resistant with other clinical colleagues to solicit expert opinions on what the underlying resistance mechanism(s) might be (Table S1, available as Supplementary data at *JAC-AMR* Online). With increased global popularity of this approach among clinicians, we sought to expand upon the five main principles of this approach by engaging a panel of experts to draft a comprehensive review.

## Step 1. Collaborate with the clinical microbiology laboratory to confirm organism identity and antimicrobial susceptibility test (AST) methods

AST is one of the most important functions of the clinical microbiology laboratory. While multiple AST methods are available, demonstrating MICs via automated susceptibility test methods is most commonly used by clinical laboratories.^12^ Identification of resistance mechanisms can be exceptionally challenging despite the availability of a multitude of test options, and it is unlikely that any one test method will screen for and confirm a resistance phenotype (Table 1). Often, laboratories will use supplemental test methods in conjunction with automated AST to detect subtle resistance mechanisms.^12^ Nonetheless, a tremendous amount of valuable information can be gleaned from the MICs obtained from automated AST, and various approaches can be used to detect resistance patterns or phenotypes.

**Table 1.** Examples of major resistance mechanisms, testing methods and considerations

Resistance mechanism category	Interpretative reading		Confirmatory testing		Confirmatory test options
	Pros	Cons	Pros	Cons	
All mechanisms	Useful for the detection and characterization of low-level resistance produced by a single enzyme, that may be missed by traditional doubling dilution susceptibility test methods.^13^ May offer universal insight into resistance mechanism regardless of differences in interpretive criteria (breakpoints).^13^	The presence of multiple resistance mechanisms can make antibiotic resistance patterns misleading.^14^ Correct identification of the organism to the species level is required.^13^ In some cases, the level of resistance to an antibiotic (and detectability) may be regulated by the degree of enzyme production, making interpretative reading less reliable.^15^ Requires that an inclusive range of antibiotics are tested, even if some drugs are not obvious therapeutic choices.^13,15^	Due to variability in reliability of interpretative methods, confirmatory tests may be needed to accurately identify the presence of resistance mechanisms, particularly ESBLs and CREs.^16^ Supports infection prevention and public health surveillance efforts.^13^ Molecular methods, if available, are highly sensitive and confirmatory.^16^	For some tests, interpretation may be challenging and require staff with extensive training and expertise.^17^ Relying on MICs and using lowered cephalosporin breakpoints is a reliable option for clinical care, while phenotypic confirmatory testing may not be additive and is a strain on laboratory resources.^17^	See individual resistance mechanisms
ESBL	Ceftazidime and/or cefpodoxime are reliable indicator drugs for most of the TEM- and SHV-derived ESBLs.^14^ Cefotaxime may serve as a more helpful indicator drug for CTX-M ESBLs.^14^ Resistance to ceftazidime in the absence of resistance to cefoxitin is a reliable screening test and highly suggestive of ESBL.^14^	Isolates with very small or very large amounts of enzyme may not behave predictively, particularly with BLI combination drugs.^15^	Use of MICs from indicator drugs (e.g. ceftriaxone MIC of ≥2 mg/L are sensitive but not specific. Confirmatory tests are needed to accurately identify the presence of ESBLs.^18^ May be used to identify ESBLs in organisms not frequently associated with ESBLs (e.g. *Citrobacter* species*, Serratia* species).^18^	The presence of AmpC cephalosporinases renders ESBL confirmatory tests unreliable.^17^ Molecular methods are limited to select ESBL types.^18^ Supplemental ESBL testing is only approved for *E. coli*, *K. pneumoniae*, *Klebsiella oxytoca*, *Proteus mirabilis.*^19^	Double disc synergy test (phenotypic).^20^ Gradient diffusion (phenotypic, ESBL strip).^20^ Combined disc test (cephalosporin and clavulanate, phenotypic).^20^ Gene detection (molecular).^21^ Chromogenic media (phenotypic).^15,22^
AmpC β-lactamase	In Enterobacterales, resistance to cefoxitin without cross-resistance to oxyimino cephalosporins is nearly diagnostic for inducible AmpC.^14,15^ With the exception of inhibition of the AmpC enzyme produced by *Morganella morganii* with tazobactam, the inability of BLIs to inhibit is suggestive of derepressed AmpC production in Enterobacterales.^14^	Not all AmpC producers are resistant to cefoxitin. Some organisms may demonstrate susceptibility due to lack of permeation of porins.^23^	May be able to help differentiate from other mechanisms, particularly in the case of cefoxitin susceptibility.^24^	Technically challenging and time consuming, some may not be reliable enough for regular use in the clinical microbiology laboratory. Slow turnaround time, variable performance.^24^	Disc potentiation test (phenotypic).^24–26^ Double disc synergy test (phenotypic).^24–26^ Modified 3D tests.^24^
CRE	Relatively straight-forward, resistance to one or more carbapenems implies a carbapenem-resistant organism, possibly producing a carbapenemase enzyme.^14,27^	Interpretation of MIC patterns does not reliably differentiate organisms with ESBL or AmpC and porin loss or deficiency (non-carbapenemase-producing CRE) versus carbapenemase-producing CRE.^27–29^	Timely and accurate identification of carbapenemase-producing CRE enables prompt infection prevention measures.^29^ Differentiation between carbapenemase-producing CRE and non-carbapenemase-producing CRE allows for treatment stratification and better guidance of clinical management.^30^	Turnaround time may be limited by testing sent out to reference labs or public health labs for confirmatory testing in centres where in-house confirmation is not performed.	Colorimetric assay for detecting carbapenem hydrolysis (phenotypic).^19^ EDTA-modified carbapenem inactivation method or modified carbapenem inactivation method (phenotypic).^19^ Nucleic acid-based tests (genotypic).^19,29^

CRE, carbapenem-resistant Enterobacterales.

‘Interpretative reading’ is a core methodology employed by clinicians and involves analysing the susceptibility pattern of a given organism, not just the results of individual antibiotics.^13,14,31^ Commercially available automated test systems employ different variations of what are known as ‘expert test systems’. These systems are computers that use data to simulate human decision-making and use facts to infer what is already known.^32^ These systems offer significant advantages such as the capacity to hold large amounts of data, reduce human error and provide consistent answers for repetitive decisions. Often, the algorithms built into the expert system are based on Boolean (if/then) logic, allowing the computer to use MIC distribution data to make interpretive decisions based on accepted criteria such as those from the CLSI,^19^ EUCAST^33^ or published literature.^32^ While some systems contain data on thousands of phenotypes and MIC distributions, it is important to note that the system’s performance depends on the quality and depth of the domain knowledge within the expert system database. While these systems may be capable of screening for known resistance patterns, they may be less likely to detect subtle mechanisms for which there are limited data, when there are multiple resistance mechanisms present, or when there is low-level resistance to certain antimicrobials.^32^

Using confirmatory tests for common resistance mechanisms is controversial and variable, depending on the mechanism. For ESBL-producing Gram-negative organisms, proponents of confirmatory testing argue that MICs alone are insufficient to identify them effectively.^18^ For example, a ceftriaxone MIC of >2 mg/L may be sensitive for the detection of an ESBL in a *Klebsiella* species. Still, it is not specific since not all *Klebsiella* species with a ceftriaxone MIC of >2 mg/L are ESBL producers as this method also detects AmpC and carbapenemases.^18^ From a clinical perspective, using MICs alone could overestimate the prevalence of ESBLs and encourage excessive carbapenem use.^18^ On the contrary, interpretative reading may be more effective than confirmatory testing to detect β-lactamases. Confirmatory tests for AmpC production are limited, but AmpC-producing organisms are consistently resistant to the cephamycin class of antibiotics (e.g. cefoxitin), setting them apart from the resistance profile of their ESBL counterparts.^16^ Importantly, both interpretative reading and confirmatory methods may become misleading when organisms harbouring more than one resistance mechanism are tested (Table 1).^14^

## Step 2. Acknowledge bacterial-specific mechanisms of intrinsic resistance

A critical component of AMR evaluation is familiarity with intrinsic pathogen resistance and its implication on antimicrobial susceptibility. Both Gram-positive and Gram-negative microorganisms possess diverse means by which they may evade antimicrobial activity, with resistance described as intrinsic, induced or acquired.^34^ Intrinsic resistance refers to resistance displayed universally at baseline within a bacterial species, independent of exposure to antibiotics or transfer of genetic material.^35^ Common mechanisms that contribute to intrinsic resistance include outer structure permeability, efflux pump activity, target site affinity and drug inactivation.

Intrinsic barriers limiting antimicrobial access to target cell mechanisms include passive restriction due to cell permeability or active drug removal via efflux mechanisms. While the Gram-positive peptidoglycan layer is estimated to have a large permeability threshold, allowing passage of molecules 30–57 kDa,^36^ the tightly-bound LPS membrane in Gram-negative organisms generally restricts penetration of molecules exceeding 600 Da,^37^ and those that are lipophilic in nature, thus imparting natural resistance to antimicrobials such as clindamycin, glycopeptides and oxazolidinones.^38^ Molecular passage through the Gram-negative membrane primarily relies on diffusion through embedded porin channels, wherein alterations such as porin reduction or mutational changes in selectivity may decrease antimicrobial transport.^39^ For example, the high intrinsic resistance of *Acinetobacter* species is partially attributed to its outer membrane impermeability and diminished porin expression.^40^ Efflux pumps scattered throughout the cell membrane contribute to cellular homeostasis and the removal of solutes such as antimicrobials. Efflux pumps may be specific towards a single compound, a class of antimicrobials (e.g. tetracycline resistance via various Tet efflux pumps such as Tet(A) or even multiple classes of antimicrobials (e.g. MexAB-OprM mediated resistance to β-lactams, quinolones, tetracyclines and macrolides in *Pseudomonas aeruginosa*).^41–43^ Even with successful penetration of bacterial cell defences, alterations in target site affinity or antibiotic mechanisms may preclude drug activity. Intrinsic examples of resistance due to poor target site affinity are well described in Gram-positive organisms, such as PBP5-mediated cephalosporin resistance in *Enterococcus* species.^44^ Finally, intrinsic drug inactivation most commonly involves enzymatic activity, with β-lactamases serving as the most prevalent and clinically significant resistance method, though other mechanisms, such as aminoglycoside-modifying enzymes, also exist. While these mechanisms may also be induced or acquired, intrinsically expressed β-lactamases exist, with notable examples including *bla*_SHV-1_ expression in *Klebsiella pneumoniae* resulting in baseline ampicillin resistance, as well as the widespread resistance of *Stenotrophomonas maltophilia* to the β-lactam class as a whole via β-lactamases *bla*_L1_ and *bla*_L2_.^45–47^

Ultimately, antimicrobials associated with intrinsic resistance are generally either excluded from antibiogram susceptibility reporting or summarized as resistant for organisms known to express intrinsic resistance to such agents, often supported by helpful reference tables and guidance provided by organizations such as CLSI and EUCAST.^19,33^ Table 2 outlines commonly encountered organisms and expected intrinsic resistance mechanisms, recognition of which is an essential asset of any clinician navigating the art of antimicrobial selection.

**Table 2.** Bacterial intrinsic resistance mechanisms and antimicrobial susceptibility

Organism	Mechanism of intrinsic resistance (enzyme or gene)	Antimicrobial agent
*Staphylococcus aureus* ^48,49^	Efflux pump (NorA) β-Lactamase (*bla*_Z_) Target site (*mecA*)	Fluoroquinolones Penicillin Methicillin, oxacillin
*Enterococcus* spp.^45^	Target site (PBP5) Target site^a^ (*vanA*, *vanB*, *vanC*)	Cephalosporins Vancomycin
*E. coli* ^50^	Impermeability Efflux pump (AcrAB-TolC) Target site	Glycopeptides, clindamycin Oxazolidinones Daptomycin
*Proteus* spp.^42,51–53^	Impermeability Efflux pump (*tet*) Target activity (*nfsA*, *nfsB*) Target site (arnBCADTEF, *eptB*)	Glycopeptides, clindamycin Tetracyclines Nitrofurantoin Colistin, polymyxin B
*K. pneumoniae* ^45,54^	Impermeability β-Lactamase (*bla*_SHV-1_) Enzymatic inactivation (*fosA*)	Glycopeptides, clindamycin Ampicillin Fosfomycin
*Providencia* spp.^53,55^	Target site (arnBCADTEF, *eptB*) Efflux pump (AcrAB)	Colistin, polymyxin B Tigecycline
*E. cloacae* ^56^	β-Lactamase (AmpC)	Ampicillin ± sulbactam, amoxicillin ± clavulanate, first-generation cephalosporins, cephamycins
*P. aeruginosa* ^57,58^	Impermeability (OprF) Efflux pump (MexAB-OprM, MexXY-OprmM) β-Lactamase (AmpC)	Widespread low antimicrobial permeability β-Lactams, quinolones, tetracyclines, macrolides Ampicillin ± sulbactam, amoxicillin ± clavulanate, first-generation cephalosporins, cephamycins
*S. maltophilia* ^46,47,59^	β-Lactamase (*bla*_L1_) β-Lactamase (*bla*_L2_) AME [AAC(6′)-Iz, AAC(6′)-Iak, Aph(3′)-IIc]	Penicillins, cephalosporins, carbapenems Penicillins, cephalosporins, aztreonam Aminoglycosides
*Acinetobacter* spp.^60–62^	Impermeability (OmpA_ab_) Efflux pump (AdeABC) β-Lactamase (*bla*_TEM-1_, *bla*_SHV-1_) β-Lactamase (AmpC) β-Lactamase (OXA-69, OXA-51)	Widespread low antimicrobial permeability β-Lactams, aminoglycosides, tetracyclines, trimethoprim/sulfamethoxazole, quinolones Penicillins, first-generation cephalosporins Ampicillin ± sulbactam, amoxicillin ± clavulanate, cephalosporins Oxacillin, carbapenems (low level)

AME, aminoglycoside-modifying enzyme.

a Intrinsic resistance specific to *Enterococcus casseliflavus*, *Enterococcus flavescens and Enterococcus gallinarum* species.

## Step 3. Review local, regional and geographical epidemiology of AMR

It is important to understand the epidemiology of antibiotic resistance to help determine the relative risk of a resistant pathogen in a given patient. Several methods are used to track antibiotic resistance at both local and global levels.

The AST of isolates cultured from patient specimens is usually performed with several antimicrobial agents to determine an antibiogram, or a summary of resistance patterns amongst pathogens isolated at a given medical centre. Periodically (at least annually in most centres), the microbiology laboratory will compile cumulative susceptibilities, which the clinical team can use to help guide empirical therapy choices.^63^ Additionally, this susceptibility snapshot provides information about local epidemiology and resistance trends and, in addition to supporting clinical decision-making, may help the microbiology laboratory identify unusual resistance patterns when they arise. One shortcoming of local antibiograms is that they depend on the laboratory methods for determining susceptibility. Many commercially available automated test systems use dilution ranges around the breakpoint for a given drug and may not provide information that is granular enough to discriminate between resistance mechanisms.^64^ Furthermore, resistance breakpoints used clinically for detecting acquired resistance determinants may not coincide with therapeutic breakpoints used in the clinical microbiology laboratory. Nevertheless, generating an antibiogram is technically easy to do and interpret, even in small and resource-limited laboratories. It is a relatively low-cost activity suitable for testing large numbers of isolates and relies on routine clinical practice with good reproducibility.^65^

**Figure 2.**
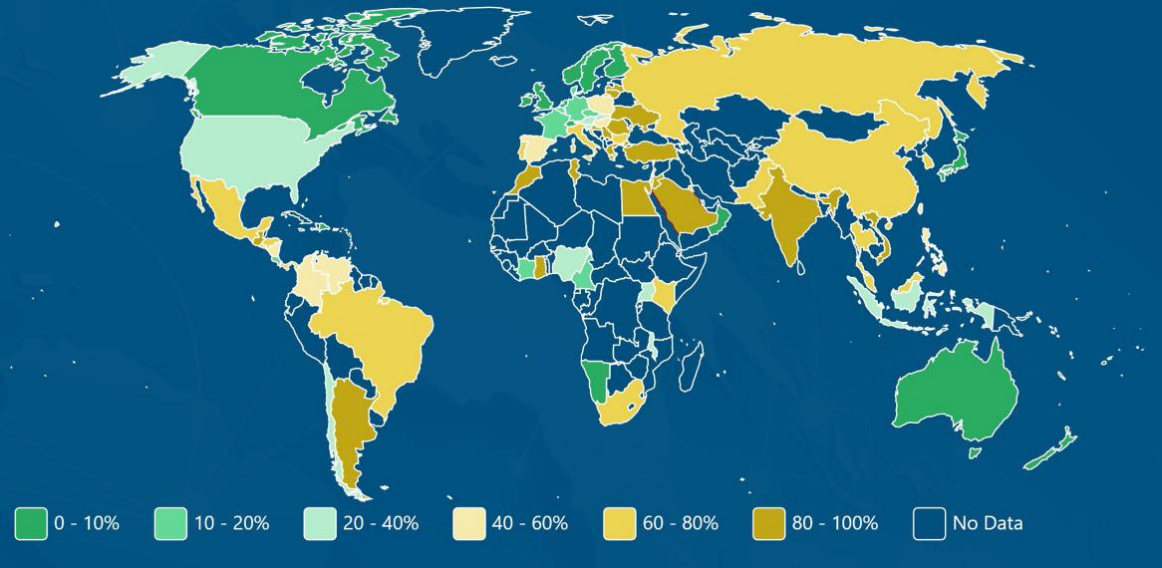
Heatmap of global prevalence of carbapenem-resistant *Acinetobacter* spp. from Antimicrobial Testing Leadership and Surveillance (ATLAS). Generated from https://atlas-surveillance.com/#/heatmap/resistance.

Beyond what is generated locally, there are several resources of large-scale, often global, datasets for AST information (Table 3). These data sources differ in their collection methods and available data level. For example, the Global Antimicrobial Resistance and Use Surveillance System (GLASS) provides data summaries in graphical and tabular formats, whereas raw datasets of line listing of MIC values by organism are available through Vivli’s AMR register. In addition, many of the resources listed in Table 3 have searchable databases that can be used to generate figures and tables of pathogens and antibiotics of interest. For example, the global prevalence of carbapenem-resistant *Acinetobacter* spp. ranges from <10% in Canada and Australia to >80% in India and Argentina (Figure 2). Even this country-level data can help a treating physician to understand the relative risk of a particular resistance in their locale and make informed choices regarding appropriate antibiotic selection for empirical therapy.

**Table 3.** Sources of freely accessible antimicrobial susceptibility surveillance data

Name of programme	Owner	Contents	URL
Surveillance Atlas (formerly EARRS-net)	ECDC	Heat maps of resistance for European countries. Searchable database.	http://atlas.ecdc.europa.eu/public/index.aspx
GLASS	WHO	Graphical representations of AST usage and antimicrobial resistance.	https://worldhealthorg.shinyapps.io/glass-dashboard/_w_cdaa8770/#!/amr
Antimicrobial WT distributions of microorganisms	EUCAST	MIC frequency distribution and epidemiological cut-offs (ECOFFs).	https://mic.eucast.org/search/
AMR Register	Vivli	Raw datasets contributed by pharmaceutical companies from their surveillance programmes. Searchable database.	https://amr.vivli.org/
SENTRY MVP	JMI Laboratories	Global surveillance data presented as MIC distributions or heat maps. Searchable database. USA data available by census region.	https://sentry-mvp.jmilabs.com/
ATLAS	Pfizer	Global surveillance data presented as MIC distributions or heat maps. Searchable database.	https://atlas-surveillance.com
Study for monitoring antimicrobial resistance trends (SMART)	Merck	Heatmaps of resistance. Searchable database.	https://globalsmartsite.com
Keystone	Paratek	Global surveillance data presented as MIC distributions or heat maps. Searchable database. USA data available by census region.	https://paratek-keystone.com/

Antibiograms define the phenotypic expression of resistance patterns in bacterial isolates. However this may not be sufficient in understanding strain-relatedness. Molecular typing methods have become important in examining the spread of specific resistance determinants and determining if a particular strain is endemic or causing an outbreak.^66^ Currently, next-generation sequencing (NGS) technology is an easy and cost-effective tool for performing molecular epidemiology by sequencing the entire genome of pathogens of interest. MLST utilizes nucleotide sequencing to detect variations in fragments of 5 to 10 housekeeping genes, with a single point mutation difference between genes considered to be a new allele.^67^ Previously conducted by the PCR method, MLST is now routinely performed by WGS, with the examination of the various loci as the standard methodology.^68,69^ The MLST data can be shared and tracked across laboratories via several websites, like http://pubmlst.org and https://bigsdb.pasteur.fr/. Examples of the utility of WGS include investigating a prolonged outbreak of *K. pneumoniae* carbapenemase (KPC)-producing *K. pneumoniae* and *Enterobacter cloacae* in a burn unit in the USA that, at face value, appeared epidemiologically unrelated but were found to be genetically linked.^70^ WGS was also used to track an outbreak of carbapenem-resistant *K. pneumoniae* expressing OXA-232 to a contaminated duodenoscope in a California hospital.^71^

Regardless of the methods used, understanding the epidemiology of resistance is critical to patient care. Although resistance patterns may be geographically distinct, the threat remains that any new resistance mechanism may spread rapidly to other areas. For example, the gene encoding NDM 1 (*bla*_NDM-1_) leading to resistance to most β-lactam antibiotics was identified as early as 2008.^72^ Its occurrence initially was confined to the Indian subcontinent.^72,73^ However, the gene became globally disseminated in less than 5 years of the first known occurrence, partly due to the travel of infected or colonized individuals to Europe, the Middle East and North America.^74^ Thus, it is important to monitor resistance as a function of time and location to use this knowledge to inform antimicrobial stewardship.

## Step 4. Understand acquired resistance mechanisms and interpretive antibiogram reading

Bacterial organisms evade antibiotics by supplementing their intrinsic AMR with acquired resistance mechanisms. Acquired resistance occurs through the horizontal gene transfer (HGT) modalities of transformation (free DNA uptake), transduction (phage vector genetic exchange), and conjugation (cell-to-cell movement of genetic elements).^75^ Conjugation dominates resistance development in clinical pathogens through mobile genetic elements (MGEs), such as transposons and narrow/broad host-range plasmids.^76^ A prime example is the plasmid-mediated dissemination of β-lactamase (*bla*) genes among Enterobacterales. Acquired AMR to β-lactams in Enterobacterales and *P. aeruginosa* is multifactorial and includes: (i) modification/inactivation by β-lactamases; (ii) reduced uptake due to porin changes; (iii) increased elimination through efflux pumps; and in rare cases (iv) modifications to target PBP.^77^ The complex interplay of these AMR mechanisms and limited clinical microbiological information make bedside susceptibility interpretation and treatment selection challenging. Therefore, separating a few important AMR mechanisms found in *Escherichia coli*, *K. pneumoniae* and *P. aeruginosa* into individual components may help clarify their phenotypic impact when analysing a clinical isolate (Table 4).

**Table 4.** AST results (MicroScan Walkaway, Beckman Coulter, Indianapolis, IN, USA)

Antimicrobial	MIC (μg/mL)	Interpretation
Amikacin	>16	R
Ampicillin/sulbactam	>32/8	R
Aztreonam	>16	R
Cefazolin	>16	R
Cefepime	>16	R
Ceftazidime/avibactam	>16/4	R
Ceftriaxone	>4	R
Colistin		I
Ciprofloxacin	>2	R
Ertapenem	>2	R
Gentamicin	>8	R
Meropenem	>8	R
Meropenem/vaborbactam	>16/8	R
Piperacillin/tazobactam	>64/4	R
Tobramycin	>8	R

All interpretations are consistent with the CLSI M100 as of April 2023. I, intermediate; R, resistant; S, susceptible.

ESBLs, belonging to the Ambler Class A serine-β-lactamases, are prevalent globally in *E. coli* and *K. pneumoniae* pathogens, predominated by the cefotaximase-Munich (CTX-M) family that originated from the *bla* genes present in *Kluyvera* species, which are predominantly found as part of the human normal intestine microbiome.^78,79^ The presence of a CTX-M-15 ESBL in these organisms results in phenotypic resistance to penicillins (PENs), early cephalosporins (E-CEPHs), expanded-spectrum cephalosporins (ES-CEPHs) and aztreonam through hydrolysis of these β-lactams. Susceptibility is retained for cephamycins (CMCs) such as cefoxitin and cefotetan, carbapenems (CARBs) and contemporary β-lactam/β-lactamase inhibitor (BL/BLI) combinations such as ceftazidime/avibactam and meropenem/vaborbactam. The treatment of these ESBL organisms with the drug of choice, carbapenems, may select for carbapenem-resistant (CR) Enterobacterales with additional resistance mechanisms of note.

Some CR *E. coli* and *K. pneumoniae* produce carbapenemases such as NDM-1, KPC and oxacillinase-48-like (OXA-48-like) enzymes. The phenotypic profiles of these carbapenemase-producing organisms are unique and can be useful for clinical interpretation when carbapenemase phenotypic or genotypic testing is unavailable. The presence of NDM-1 alone can be suspected phenotypically when aztreonam retains activity while resistance to all other available β-lactams is seen, including contemporary BL/BLIs such as ceftazidime/avibactam, meropenem/vaborbactam and imipenem/relebactam.^80–82^ Cefiderocol, and pipeline agents like aztreonam/avibactam, also retain activity against most NDM-1-producing isolates, but clinical AST may not be readily available or timely.^83–85^

Alternatively, KPC may be suspected in cases where broad β-lactam resistance includes high-level resistance to CARBs and aztreonam, but susceptibility to ceftazidime/avibactam, meropenem/vaborbactam and imipenem/relebactam remains.^86^ Meropenem/vaborbactam tends to have a lower MIC for KPC isolates than ceftazidime/avibactam.^87,88^ This additional phenotypic clue can be helpful when deciding definitive therapy since resistance development has been more commonly reported with ceftazidime/avibactam than meropenem/vaborbactam.^89,90^

Lastly, organisms producing OXA-48-like carbapenemases are resistant to PENs and typically other β-lactams such as E-CEPHs, ES-CEPHs and CARBs; however, phenotypic analysis is difficult due to variability among different OXA variants.^91,92^ When resistance to meropenem and imipenem is seen in OXA-48-like-producing organisms, the addition of contemporary BLIs like vaborbactam and relebactam does not restore susceptibility as neither inhibitor can effectively block the enzyme.^93^ Ceftazidime/avibactam susceptibility remains in these organisms because avibactam can inhibit OXA-48-like hydrolysis of ceftazidime. Therefore, an OXA-48-like producer resistant to CARBs will often show phenotypic susceptibility to ceftazidime/avibactam but retain resistance to meropenem/vaborbactam and imipenem/relebactam. This contrasts with a KPC producer, where susceptibility to all three contemporary BL/BLIs is likely, and an NDM-1 producer, which would show resistance to all three.

In addition to β-lactamases, porin and efflux mutations are important acquired AMR mechanisms against β-lactams, particularly exemplified by *P. aeruginosa*.^94,95^ OprD mutations may phenotypically manifest as imipenem resistant, meropenem variable and susceptible to non-carbapenem antipseudomonal β-lactams like piperacillin/tazobactam, cefepime and ceftazidime. Conversely, mutations leading to MexAB-OprM efflux up-regulation will have a nearly flipped profile where susceptibility to imipenem remains. In contrast, meropenem and the non-carbapenem antipseudomonal β-lactams can have elevated MICs or resistance.

It is important to note that the AMR mechanisms presented in this review are not exhaustive and describe typical phenotypic findings when analysing one specific resistance mechanism. Unfortunately, clinical isolates often concurrently possess multiple acquired AMR mechanisms (concomitant/mutated β-lactamases, porin/efflux changes, increased gene copy number etc.).^96,97^ This makes phenotypic analysis extremely complex, especially in the absence of confirmatory genotypic testing.

## Step 5. Putting it all together to treat a clinically relevant infection

**Figure 3.**
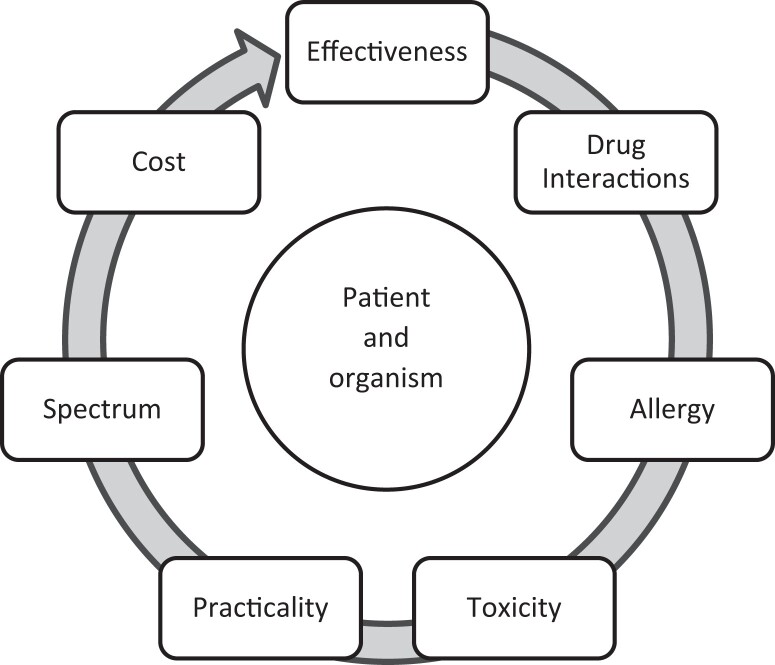
Antimicrobial decision-making heuristic.

Once the organism susceptibility testing and resistance profile have been released, this information must be integrated with relevant clinical literature, societal and institutional clinical guidelines^4,98–100^ and patient-specific factors to identify the optimal treatment plan. As there are usually multiple potential antimicrobial choices, several key factors should be accounted for in any decision-making heuristic. These are described in Figure 3. First and foremost among these is the likely clinical effectiveness of an antimicrobial for the organism at hand at a given site of infection. In certain cases, randomized controlled trial data may address a specific question of interest, as with piperacillin/tazobactam versus meropenem for third-generation cephalosporin-resistant *E. coli* and the MERINO trial^101^ or the ZEPHyR trial for linezolid versus vancomycin for MRSA pneumonia.^102^ More commonly, however, assessments must be made based on observational data (e.g. linezolid versus daptomycin for VRE bacteraemia^103,104^) or subgroups of randomized trial data (e.g. daptomycin versus vancomycin for MRSA bacteraemia^105^ or ceftolozane/tazobactam for ESBL-producing Enterobacterales^106^). In less common circumstances, potential treatments are assessed using *in vitro* data with or without clinical case data (e.g. oritavancin for daptomycin- and linezolid-resistant VRE infections^107,108^).

Rarely, these assessments may essentially ‘rule out’ a potential treatment option, as has been seen with randomized and observational comparisons of contemporary β-lactam/β-lactamase inhibitor combinations versus best available therapy.^109–111^ Next, drug interactions with necessary medications (e.g. carbapenems with valproic acid)^112^ or allergies may eliminate certain antimicrobials as therapeutic options. More commonly, however, a seeming clinical equipoise is established, and other factors must be considered. The relative weight of these factors may vary based on patient- and setting-specific considerations. In certain cases, potential toxicity may rule out a treatment. For example, definitive aminoglycoside therapy may be appropriate for pyelonephritis caused by an ESBL-producing organism in an otherwise healthy individual. In contrast, carbapenem therapy may be preferred in an older patient prone to nephrotoxicity.^113,114^ In other situations, a perfectly suitable option for inpatient use may be impractical or unsafe for home use. An equally effective alternative with a more favourable toxicity profile may be substituted to facilitate discharge (e.g. daptomycin or oral linezolid versus vancomycin for severe MRSA infections). Other considerations are perhaps more subjective and value-based, including institutional and individual cost, the spectrum of activity, and the likelihood of selection of AMR. These may vary significantly based on setting and individual preferences. For example, a change from cefepime to ceftriaxone for pyelonephritis caused by cefazolin-resistant *E. coli* may be desired to reduce unnecessary exposure to antipseudomonal antibiotics,^115^ but for an individual colonized with *Clostridioides difficile* this may increase the likelihood of *C. difficile* infection (CDI).^116,117^

Key to these considerations is the placement of the patient at the centre of the decision-making process. The organism susceptibility profile may determine a starting point for this process, but subsequent decisions should be made to provide the patient with the most effective, safe and appropriate antimicrobial.

A case can highlight each principle of AMRrounds and arrive at a final, rational treatment decision. For example, consider a patient transferred to a US hospital from a hospital in Yemen following initial treatment for perforated appendicitis with subsequent surgical complications. A limited transfer summary contains minimal microbiological history but notes that a blood culture obtained 4 days before transfer was positive for *E. coli*. You are called 3 days later when an admission blood culture results with a meropenem-resistant *E. coli* (Table 4) and are asked to provide treatment recommendations. A medical record review notes that the patient is febrile but generally clinically stable. An abdominal CT reveals a 5 × 6 cm fluid collection in the right lower quadrant. A urine culture collected on admission was positive for an *E. coli* with the same phenotype.

### Step 1. Collaborate with the clinical microbiology laboratory to confirm organism identity and AST methods

A call to the microbiologist confirms that confirmatory testing is being performed for meropenem, meropenem/vaborbactam and ceftazidime/avibactam using gradient diffusion strips. A CARBA 5 phenotypic assay (Hardy Diagnostics, Santa Maria, CA, USA) has been performed per usual laboratory practice for any carbapenem-resistant Enterobacterales from a sterile site.

### Step 2. Acknowledge bacterial-specific mechanisms of intrinsic resistance

*E. coli* is known to harbour a non-inducible AmpC β-lactamase that is expressed constitutively at a low level.^118^ This, is, however, an uncommon contributor to AMR in clinical isolates and would not explain the carbapenem-resistant phenotype.^119^ Thus, alternative explanations beyond intrinsic resistance are necessary to adequately describe the resistance phenotype of this isolate.

### Step 3 Review local, regional and geographical epidemiology of AMR

A PubMed search using the string ‘Yemen and carbapenem-resistant’ yields two papers describing the epidemiology of carbapenem resistant Enterobacterales at hospitals in Yemen.^120,121^ These papers describe a high prevalence of *bla*_NDM_, *bla*_OXA-48-like_ and ESBL enzymes in clinical carbapenem-resistant Enterobacterales.

### Step 4. Understand acquired resistance mechanisms and interpretive antibiogram reading

The degree of resistance present in this isolate and the limited molecular data available from Yemen isolates provide valuable information. First, meropenem resistance, alongside ceftazidime/avibactam and meropenem/vaborbactam resistance, is consistent with the presence of an NDM enzyme. Isolates solely harbouring NDM often retain susceptibility to aztreonam. Hence, in this case, the resistance suggests that one or more additional β-lactamases, such as an OXA-type or ESBL enzyme, may be present.^82,122^ High-level aminoglycoside resistance may be explained by the presence of ribosomal modification enzymes, which are often found on NDM-harbouring plasmids.^123,124^ Thus, this susceptibility profile is consistent with an MBL enzyme, and likely one or more additional serine-β-lactamases. The CARBA 5 assay performed on the patient identified the presence of both NDM-type and OXA-48-like enzymes.

### Step 5. Putting it all together to treat a clinically relevant infection

Understanding that the patient has bacteraemia with an organism producing both NDM and OXA-48-like enzymes and the phenotypic susceptibility profile provides a starting point for antimicrobial selection. A literature review indicates that aztreonam/avibactam, cefiderocol, colistin and the tetracycline derivatives tigecycline and eravacycline are likely to have *in vitro* activity against such isolates.^82,125,126^ Clinical data suggest cefiderocol or a combination of ceftazidime/avibactam with aztreonam are effective treatment options compared with alternative agents,^84,110^ and societal guidance supports these agents as appropriate therapies for infections caused by *E. coli* harbouring NDM enzymes.^127^ No patient-specific factors are present that would favour one agent over another, so institutional factors, such as local availability, ability to perform susceptibility testing, or clinician preference would dictate antimicrobial decision-making.

### Conclusions

Even in an era of considerable advances in laboratory diagnostics, AMR remains an intriguing challenge for clinicians. This review presents a detailed discussion on one systematic approach that poses a reasonable starting point for both the clinical management of patients suffering from AMR-related infections and educating clinicians on AMR in general. The methods detailed throughout this review should also serve as a useful comprehensive and standardized approach to addressing AMR within the context of ASPs. We propose that clinicians, educators and researchers will benefit from this new scheme that uses a foundation of mechanistic phenotypes that should help facilitate clinical reasoning and enhance comparability among clinical studies that inform care. Future research is needed to build upon the effectiveness of these principles and further explore the accuracy of this approach to understand if more generalizable conclusions can be drawn.

## Supplementary Material

dlad097_Supplementary_Data

## Supplementary data

Table S1 available as Supplementary data at *JAC-AMR* Online.
