# Supplementary material for: #AMRrounds: a systematic educational approach for navigating bench to bedside antimicrobial resistance
Source: JAC Antimicrob Resist. 2023 Aug 14;5(4):dlad097. doi: 10.1093/jacamr/dlad097 (PMC10424884; doi:10.1093/jacamr/dlad097)
Supplement: dlad097_Supplementary_Data [file dlad097_supplementary_data.docx]

**Table S1. #AMRrounds on Twitter**

| **Twitter #AMRrounds – Example Threads** |
| --- |
| 5/15/22 – Serratia marcescens  <https://twitter.com/wfwrighid/status/1525816003405434882?s=46&t=a4arOA1s55xpOtPWvkSN3A> |
| 7/9/2022 – Citrobacter freundii  <https://twitter.com/wfwrighid/status/145833815628734465?s=46&t=a4arOA1s55xpOtPWvkSN3A> |
| 8/17/2022 – Pseudomonas aeruginosa  <https://twitter.com/wfwrighid/status/1559890885843763202?s=46&t=a4arOA1s55xpOtPWvkSN3A> |
| 11/28/2022 – Escherichia coli and Klebsiella pneumoniae complex  <https://twitter.com/wfwrighid/status/1597255128863494146?s=46&t=a4arOA1s55xpOtPWvkSN3A> |
| 11/30/2022 – Acinetobacter baumannii/calcoaceticus complex  <https://twitter.com/wfwrighid/status/1598030838406713344?s=46&t=a4arOA1s55xpOtPWvkSN3A> |
| 2/8/2023 – Klebsiella pneumoniae  <https://twitter.com/wfwrighid/status/1623343826352975874?s=46&t=a4arOA1s55xpOtPWvkSN3A> |
